# Determination of Free Radical Scavenging, Antioxidative DNA Damage Activities and Phytochemical Components of Active Fractions from *Lansium domesticum* Corr. Fruit

**DOI:** 10.3390/nu7085312

**Published:** 2015-08-14

**Authors:** Prapaipat Klungsupya, Nava Suthepakul, Thanchanok Muangman, Ubon Rerk-Am, Jeerayu Thongdon-A

**Affiliations:** 1 Department of Pharmaceuticals and Natural Products, Thailand Institute of Scientific and Technological Research (TISTR), 35 Mu 3 Techno Polis, Khlong Luang, Pathum Thani 12120, Thailand; E-Mails: thanchanok@tistr.or.th (T.M.); ubon@tistr.or.th (U.R.-A.); jeerayu@tistr.or.th (J.T.-A.); 2 The Government Pharmaceutical Organization (GPO), Rama VI Road, Bangkok 10400, Thailand; E-Mail: nava.su@hotmail.com

**Keywords:** *Lansium domesticum*, long-kong, superoxide (O_2_^−•^), hydroxyl (OH^•^), hydrogen peroxide (H_2_O_2_), DNA damage, comet test

## Abstract

*Lansium domesticum* Corr. or “long-kong” is one of the most popular fruits in Thailand. Its peel (skin, SK) and seeds (SD) become waste unless recycled or applied for use. This study was undertaken to determine the bioactivity and phytochemical components of *L. domesticum* (LD) skin and seed extracts. Following various extraction and fractionation procedures, 12 fractions were obtained. All fractions were tested for antioxidant capacity against O_2_^−•^ and OH^•^. It was found that the peel of *L. domesticum* fruits exhibited higher O_2_^−•^ and OH^•^ scavenging activity than seeds. High potential antioxidant activity was found in two fractions of 50% ethanol extract of peel followed by ethyl acetate (EA) fractionation (LDSK50-EA) and its aqueous phase (LDSK50-H_2_O). Therefore, these two active fractions were selected for further studies on their antioxidative activity against DNA damage by hydrogen peroxide (H_2_O_2_) in human TK6 cells using comet assay. The comet results revealed DNA-protective activity of both LDSK50-EA and LDSK50-H_2_O fractions when TK6 human lymphoblast cells were pre-treated at 25, 50, 100, and 200 μg/mL for 24 h prior to H_2_O_2_ exposure. The phytochemical analysis illustrated the presence of phenolic substances, mainly scopoletin, rutin, and chlorogenic acid, in these two active fractions. This study generates new information on the biological activity of *L. domesticum*. It will promote and strengthen the utilization of *L. domesticum* by-products.

## 1. Introduction

**Figure 1:**
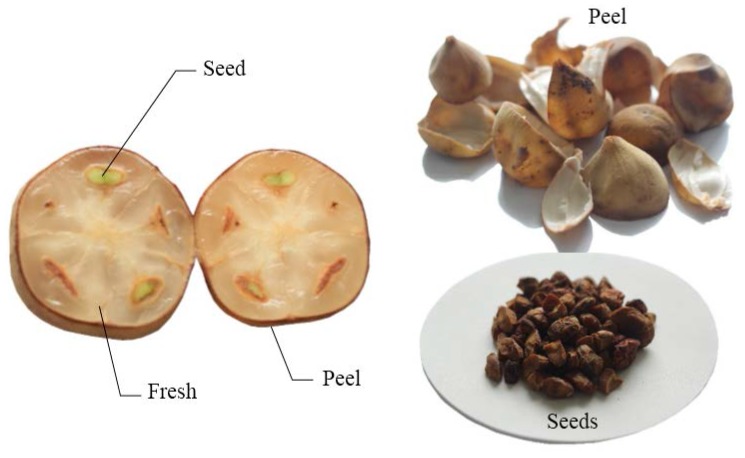
Illustration of fresh fruit, peel, and seeds of long-kong.

Thailand has a variety of fruits; however, only some of them are widely consumed. Among these is the fruit of *Lansium domesticum* Corr. which is known in Thai as “long-kong”. It has been very popular in Thailand and surrounding countries in Southeast Asia. It belongs to the Meliaceae family and is known by numerous common names. In Indonesia, it is known mainly as langsat, duku, or kokosan while in Malaysia it is known as langsat, lansa, langseh, or langsep. In the Philippines, it is known as lansones and it is known as bòn-bon in Vietnam [1,2]. The well-known and economic fruit long-kong is largely cultivated in peninsular Thailand, especially in the southern region. Long-kong develops between 15 and 25 fruits per bunch with little non-sticky sap on the skin. The appearance of long-kong fruit is globular in shape with an average size of 1.2–2.4 inches in diameter (Figure 1). It has a brittle and rough skin. It is almost seedless, with five segments of white translucent flesh [3].

The bark of *L. domesticum* is used traditionally as an anti-malarial remedy by the native people of Borneo [4]. The leaves have been used by indigenous people in the Philippines for the control of mosquitoes [5]. Previous phytochemical studies on the peels and seeds of *L. domesticum* found several types of triterpenoids [6,7]. The peel of this fruit is traditionally known to be toxic to domestic animals. Phytochemical investigations of the peels revealed the presence of triterpene glycosides and seco-onoceranoids such as lansic acid [8].

Over-production of free radical leads to “oxidative stress” that can be defined as the state of imbalance between the high production of reactive oxygen species (ROS) and the low amount of antioxidant defense systems [9]. This imbalance can cause damage to cells, contributing to cellular dysfunction and leading to chronic degenerative diseases such as atherosclerosis, diabetes, cancer, neurodegeneration, and cardiovascular diseases [10,11]. Therefore, the balance of free radical production and a sufficient level of antioxidants are essential for health [12]. Most antioxidants found in foods and supplements are of the non-enzymatic type. They boost the human enzymatic antioxidant defense system and prevent the depletion of our enzymatic antioxidants. Epidemiological evidence has supported that antioxidants have a role in the prevention of several chronic diseases including cardiovascular disease, cancer, and diabetes [13,14]. Fruits, vegetables, and medicinal herbs are the richest sources of antioxidant compounds. They are loaded with key antioxidants such as vitamin A, C, E, β-carotene, and important minerals, including selenium and zinc [15]. Moreover, the natural flavonoids (e.g., catechin, quercetin) or other phenolic (e.g., ferulic acid) or polyphenolic compounds (e.g., resveratrol) found in fruits also exert significant antioxidative ability [16].

Nowadays, the trend of using natural antioxidants has markedly increased due to the concern about the safety of synthetic antioxidants. Consequently, fruit is considered to be an important source of natural antioxidants, especially the peels and seeds which become waste unless recycled or applied to use. Even though Thailand has a variety of fruits, only some of them are widely consumed. Among these, the fruits of long-kong have been very popular in Thailand and countries in Southeast Asia. However, there is little information concerning the biological activity, particularly antioxidant activity, of peels and seeds of long-kong fruits. Therefore, this study was undertaken on long-kong to investigate the biological activities, particularly antioxidant mechanisms, using both cell-based (antioxidative DNA damage activity) and non-cell-based (ROS scavenging property) systems. Also, the phytochemical components of active fractions from *L. domesticum* Corr. fruit extracts were investigated.

## 2. Experimental Section

### 2.1. Sample Preparation and Extraction

Mature *L. domesticum* (long-kong) fruits were purchased from Talad-Thai market in Prathumthani, Thailand. After washing, the peel or skin (SK) and seeds (SD) of the fruits were separated and air-dried at 50 °C in hot air oven for 1–2 days until weight constant. Each dried sample was then ground with an electrical grinder. The grounded samples were extracted with 50% or 95% (v/v) ethanol by maceration method. Firstly, each 100 g of fine air-dried peel and seeds was mixed with 300 mL of 50% or 95% (v/v) ethanol and left overnight at room temperature. Then, supernatant of each sample was kept and added to 50% or 95% (v/v) ethanol. This step was performed 12 times to reach completion of extraction. All 12 extracts were pooled, then filtered using Whattman No.1 filter paper before being evaporated by a rotary evaporator at 45 °C to get rid of ethanol. The aqueous phase residues were further fractionated with 100 mL ethyl acetate (EA) 5 times as well as dichloromethane (DCM) of similar volume and time. All fractions were then concentrated by a rotary evaporator at 45 °C. The obtained 12 semisolid fractions were named as shown in Table 1. They were stored at 4 °C in dark conditions until utilization.

**Table 1:** Twelve fractions prepared from the peel and seeds of *L. domesticum* fruits.

Maceration	Fractionation	*Lansium domesticum* Corr.	
		Peel (SK)	Seed (SD)
Ethanol 50% (v/v) concentration	Dichloromethane (DCM)	LDSK50-DCM	LDSD50-DCM
	Ethyl acetate (EA)	LDSK50-EA	LDSD50-EA
	Water (H_2_O)	LDSK50-H_2_O	LDSD50-H_2_O
Ethanol 95% (v/v) concentration	Dichloromethane (DCM)	LDSK95-DCM	LDSD95-DCM
	Ethyl acetate (EA)	LDSK95-EA	LDSD95-EA
	Water (H_2_O)	LDSK95-H_2_O	LDSD95-H_2_O

### 2.2. Determination of ROS Radicals Scavenging Capacity

#### 2.2.1. Photochemiluminescence (PCL) Assay

The antioxidant capacity of 12 above-mentioned fractions of the peel and seeds of long-kong fruits was determined using PHOTOCHEM^®^ (Analytik Jena, Thuringia, Germany), whose principle is based upon measurement of PCL. Briefly, superoxide anion radicals (O_2_^−•^) were generated in the system by optical excitation of luminol, which was a photosensitizer substance. The antioxidant capacity of samples was measured by their inhibitory effect on luminescence generation compared with the standard antioxidant (constructed a calibration curve). The results were presented in equivalent units (nmol) of ascorbic acid for the antioxidative capacity of the water-soluble substances (ACW) system or trolox (synthetic vitamin E) units for antioxidative capacity of the lipid-soluble substances (ACL) system. For the measurement, the *L. domesticum* fractions were prepared by weighing 10 mg of each sample fraction and dissolved it in 1 mL of dilution reagent (reagent 1) supplied with the ACL or ACW reagent kits. The solution was sonicated for 10 min at room temperature to facilitate complete solubility. The supernatants were filtered through 0.45 µm syringe filter. The reaction was initiated by adding 10 µL of standard antioxidant compound (ascorbic acid and trolox) or test samples (long-kong fractions) to the mixture of 2300 µL of dilution reagent (reagent 1), 200 µL of reaction buffer (reagent 2), and 25 µL of protosensitizer (reagent 3). All samples were conducted and measured in triplicate.

#### 2.2.2. Deoxyribose Assay

Deoxyribose assay was performed to evaluate hydroxyl radical (OH^•^) scavenging activity of the 12 fractions. The method was based on the determination of malondialdehyde (MDA) pink chromogen which was a degraded product of 2-deoxyribose (2-DR) damaged by OH^•^. All sample fractions were prepared as previously mentioned in PCL assay except using distilled water as solvent. Typical reactions were started by the addition of 50 µM FeCl_3_ to solutions (0.5 mL final volume) containing 5 mM 2-DR, 100 µM ethylenediaminetetraacetic acid (EDTA), 10 mM phosphate buffer (pH 7.2), 0.5 mM H_2_O_2,_ and various concentrations of sample fractions in presence of 100 µM ascorbic acid (reducing agent) for starting the reaction and generated OH^•^. Reactions were carried out for 10 min at room temperature and stopped by the addition of 0.5 mL 2.8% trichloroacetic acid (TCA), followed by the addition of 0.5 mL thiobarbituric acid (TBA) solution. After boiling for 15 min, solutions were allowed to cool at room temperature. The absorbance of reaction mixture was measured to determine MDA pink chromogen at 532 nm in micro-plate reader system (GENios Plus, TECAN^®^, Port Melbourne, Victoria, Australia). All samples were tested in triplicate.

### 2.3. Determination of Antioxidative DNA Damage Activity by Comet Assay

#### 2.3.1. Cell Culture and Preparation

The TK6 human lymphoblasts (ATCC CRL-8015, Rockville, MD, USA) were cultured in RPMI-1640 medium (Gibco, Rockville, MD, USA) supplemented with 10% heat-activated horse serum and 1% (v/v) penicillin-streptomycin in tissue culture flask. They were maintained at 37 °C in humidified atmosphere containing 5% CO_2_ as exponential growing phase prior to the experiment. Cell density at 2 × 10^5^ cells/mL was employed for each comet assay experiment. All treatments resulted in a minimum of 70% viable cells, a level sufficient for avoiding cytotoxicity artifacts in the comet assay [17].

#### 2.3.2. Cell Treatment

After overnight culture, TK6 cells were centrifuged and the pellets were adjusted to 2 × 10^5^ cell/mL in fresh medium. One milliliter of cell suspension was added to 1 mL volumes of complete medium contained 25, 50, 100, or 200 µg/mL of LDSK50-EA (fraction of 50% ethanol extract of peel followed by ethyl acetate (EA) fractionation) or LDSK50-H_2_O (aqueous phase of 50% ethanol extract of peel followed by EA) in a 12 well-plate and incubated at 37 °C in 5% CO_2_ incubator for 24 h.

#### 2.3.3. Hydrogen Peroxide Treatment

After treatment, the chemical-containing medium was removed by centrifugation at 3500 rpm for 3 min. Cells were washed twice with cold phosphate buffered saline (PBS) before being collected by centrifugation at 3500 rpm for 3 min. The cells were resuspended in 1 mL of fresh medium containing 50 µM H_2_O_2_ and incubated at 4 °C for 5 min to produce oxidative DNA damage to the cells. At the end of incubation period, the H_2_O_2_ treated cells were washed twice with cold PBS and resuspended in cold PBS prior to subjection to comet assay.

#### 2.3.4. The Comet Assay (Single Cell Gel Electrophoresis: SCGE)

##### Comet Slide Preparation

The procedure for slide preparation performed using the standard technique was described by Singh *et al.* [18] with some modifications. Comet slides were prepared by pre-coating clean regular microscope slides with 0.75% (w/v) normal melting point (NMP) agarose. Slides were allowed to dry for 1–2 h at room temperature. The second or cell-containing layer was generally prepared from mixing 25 μL of treated cells with 75 μL of 0.5% (w/v) low melting point (LMP) agarose at 37 °C and the cell suspension was rapidly spread onto a pre-coated slide. The slides were gently covered with the coverslips and placed on a cold flat surface to allow the agarose to solidify for about 5 min. The coverslips were gently removed by sliding them sideways from the slides, and 80 μL of 0.5% LMP agarose was spread on glass slides, recovered with the coverslips and left on cold surface for agarose to solidify. At least two slides were made for each treatment.

##### Lysing, Unwinding, and Electrophoresis

The coverslips were gently removed and slides were submerged into freshly prepared lysis solution (2.5M NaCl, 100 mM EDTA, 10 mM Tris, 10% dimethyl sulfoxide (DMSO), 1% Triton X-100, pH 10; (4 °C)) for 2 h. After lysis, the slides were equilibrated in the freshly prepared electrophoresis buffer containing alkaline buffer (300 mM NaOH, 1 mM EDTA, pH > 13 at 4 °C) to allow unwinding of double-stranded DNA for approximately 20 min. The slides were then transferred into an electrophoresis unit with the same buffer and subjected to an electrophoretic field at 300 mA and 25 V at 4 °C for 20 min. The level of the electrophoresis buffer was adjusted in order to achieve 300 mA.

##### Neutralization and DNA Staining

Following electrophoresis, the slides were neutralized in 0.4 M Tris (pH 7.5) for 5 min three times. After removing the neutralization buffer, the slides were washed with cold water and allowed to dry at room temperature. The DNA was stained with 50 μL of 0.2% ethidium bromide.

##### Comet Cell Scoring

From each slide, fifty comet cells were randomly selected for comet analysis. The comet images were scored using the fluorescence microscope (at 200× magnification) connected with charge coupled device (CCD) camera. The camera was linked to a personal computer containing an automatic comet image analysis software (Comet Assay III, Perceptive Instruments, Haverhill, UK). The two parameters selected as indicator of DNA damage were tail length (TL, the distance of DNA migration measured from the center of the nucleus towards the end of the tail, µm) and tail moment (TM, a measure of the distance between the center of the tail and the center of the head, multiplied by the percentage of DNA in the tail, %).

##### Statistical Analysis

The mean values of 50 comet cells of all experiments were analyzed. All experiments were repeated on three separate occasions. The homogeneity of variance between concentration levels was determined using Levene’s test. The statistical significance of the results was determined by means of one-way analysis of variance (one-way ANOVA). When the results were significant, pair-wise comparisons of data from treated cultures with the controls were conducted using Tukey multiple comparisons. A result was considered statistically significant when the *p*-value ≤ 0.05. All analyses were performed using the SPSS statistics version 17.0 (IBM, Chicago, IL, USA).

### 2.4. Determination of Phytochemical Components

#### 2.4.1. Thin Layer Chromatography (TLC)

Stock solution containing 100 mg/mL of LDSK50-EA and 10 mg/mL of each standard was prepared by dissolving in absolute ethanol. Then, approximately 10–20 µL of LDSK50-EA stock solution and standard phytochemicals of interest (e.g., rutin, chlorogenic acid, scopoletin) were spotted on silica gel F_254_ plates Alufolien (Darmstadt, Merck, Germany). The TLC plate was developed with various solvents to select the suitable system for separation and identification.

**Solvent systems used were as the following:**

**System 1:** toluene:ethyl acetate:formic acid (5:4:1)
**System 2:** ethyl acetate:formic acid:acetic acid:water (137:11:11:26)
**Spray reagent:** natural product (diphenylboryloxyethylamine) polyethyleneglycol (PEG)
**UV Detection:** 366 nm
**Standard:** scopoletin, rutin, chlorogenic acid

The retention factor (*R*_f_ value) was used to characterize and compare components between LDSK50-EA fraction with any standard.

(1) $$\text{Retention factor }\left(R_{\text{f}}\text{ value}\right)=\frac{\text{Distance from origin to component spot}}{\text{Distance from origin to solvent front}}$$

#### 2.4.2. Total Phenolic Content (TPC) Determination

The total phenolic contents were determined by using Folin-Ciocalteu method [19,20]. The reaction mixture contained 100 µL of 2 mg/mL LDSK50-EA in ethanol, 500 µL of the Folin-Ciocalteu reagent, and 1 mL of 20% sodium carbonate. The final volume was made up to 10 mL with pure water. After 1 h incubation, the absorbance at 760 nm was measured and used to calculate the phenolic contents using gallic acid as standards. Total polyphenol contents were expressed as mg gallic acid equivalents (GAE) per mg sample extract (mg GAE/mg extract). Triplicate reactions were conducted. Data were reported as mean ± standard deviation (SD).

#### 2.4.3. Total Flavonoid Content (TFC) Determination

The total flavonoid content was determined using the aluminum chloride colorimetric method [21] with some modification. Briefly, 1 mL of the LDSK50-EA (2 mg/mL) or rutin standard solution was mixed with 5 mL of distilled water in a test tube, followed by addition of 300 μl of a 5% (w/v) sodium nitrite solution. After 5 min, 300 μl of a 10% (w/v) aluminium chloride solution was added and the mixture was allowed to stand for a further 1 min before 2 mL of 1 M NaOH was added. The mixture was made up to 10 mL with distilled water and mixed well. The absorbance was measured immediately at 510 nm. The results of triplicate analyses were expressed as mg of rutin equivalents (RE) per mg sample extract (mg RE/mg extract).

## 3. Results

### 3.1. Antioxidant Capacity of 12 Fractions of L. domesticum Extractions

#### 3.1.1. Superoxide Anion Radical Scavenging Activity

The antioxidant capacity of the 12 *L. domesticum* fractions to counteract O_2_^−•^ radicals greatly varied with the parts of *L. domesticum* extracted and the type of fractionation methods. The antioxidant capacity of each sample tested was expressed in nanomole (nmol) scale of trolox and ascorbic equivalent for the ACL and ACW substances systems, respectively. For the ACL measurement, when all samples were tested at 10 µg/mL concentration, the overall antioxidant capacity range from 0.380 to 6.625 nmol of trolox. Among the 12 fractions, it was noticeable that the LDSK50-EA possessed the highest antioxidant activity (6.625 nmol of trolox), whereas the other fractions exhibited slightly different measurements in antioxidant capacity. The degree of O_2_^−•^ scavenging activity for all 12 fractions (from high to low) were as follows: LDSK50-EA (6.625 nmol) > LDSK50-H_2_O (1.845 nmol) > LDSK95-EA (fractions of 95% ethanol extract of the *L. domesticum* skin followed by EA fractionation) (1.750 nmol) > LDSD50-EA (fractions of 50% ethanol extract of the *L. domesticum* seed followed by EA fractionation) (1.257 nmol) > LDSD95-EA (fractions of 95% ethanol extract of the *L. domesticum* seed followed by EA fractionation) (1.200 nmol) > LDSK95-H_2_O (the aqueous phase product when the *L. domesticum* skin was extracted with 95% ethanol and partitioned with EA) (1.195 nmol) > LDSK50-DCM (fractions of *L. domesticum* skin was extracted with 50% aqueous ethanol and partitioned with dichloromethane) (1.028 nmol) > LDSD95-DCM (fractions of *L. domesticum* seed was extracted with 95% ethanol and partitioned with dichloromethane) (0.966 nmol) > LDSD50-DCM (fractions of *L. domesticum* seed was extracted with 50% aqueous ethanol and partitioned with dichloromethane) (0.795 nmol) > LDSD95-H_2_O (the aqueous phase product when the *L. domesticum* seed was extracted with 95% ethanol and partitioned with EA) (0.635 nmol) > LDSD50-H_2_O (the aqueous phase product when the *L. domesticum* seed was extracted with 50% ethanol and partitioned with EA) (0.525 nmol) > LDSK95-DCM (fractions of *L. domesticum* skin was extracted with 95% ethanol and partitioned with dichloromethane) (0.380 nmol).

For the ACW measurement, the antioxidant capacity of the water-soluble system of *L. domesticum* fractions was examined at 100 µg/mL concentration. The wide range of antioxidant capacity of all fractions was found from −0.065 to 98.733 nmol of ascorbic acid. The highest antioxidant activity was in the fraction of LDSK50-H_2_O (98.733 nmol of ascorbic acid) followed by the LDSK50-EA (54.660 nmol of ascorbic acid). The overall capacity of all 12 *L. domesticum* fractions, ranked from high to low, are as follows; LDSK50-H_2_O (98.733 nmol) > LDSK50-EA (54.660 nmol) > LDSK95-H_2_O (9.910 nmol) > LDSK95-EA (8.350 nmol) > LDSD95-EA (6.880 nmol) > LDSD50-EA (5.410 nmol) > LDSK50-DCM (4.180 nmol) > LDSD50-H_2_O (2.073 nmol) > LDSD95-DCM (1.513 nmol) > LDSD95-H_2_O (1.105 nmol) > LDSK95-DCM (0.345 nmol) > LDSD50-DCM (−0.065 nmol).

#### 3.1.2. Hydroxyl Radical Scavenging Activity

The inhibitory effect of *L. domesticum* fractions on 2-DR degradation was determined by measuring the competition between 2-DR and sample fractions for the OH^•^ generated from the Fe^3+^/ascorbate/EDTA/H_2_O_2_ system. The antioxidant activity of OH^•^ scavenging was expressed as % inhibition of 2-DR degradation for the test sample of 0.5, 1.0, and 2.0 mg/mL. As shown in Table 2, the results of deoxyribose assay exhibited a wide range of OH^•^ scavenging activity, demonstrated from 0.50 ± 0.12 to 93.44 ± 0.84 in % inhibition of 2-DR degradation.

**Table 2:** Percent inhibition of 2-deoxyribose (2-DR) degradation of the 12 *L. domesticum* fractions by deoxyribose assay.

Fractions		% Inhibition (Mean ± SD)		
		0.5 mg/mL	1.0 mg/mL	2.0 mg/mL
LDSK50	DCM	20.79 ± 0.62	20.61 ± 0.87 *	43.94 ± 1.03
	EA	21.49 ± 1.28	31.24 ± 0.86	42.70 ± 0.86
	H_2_O	27.79 ± 0.54 *	71.21 ± 0.73 *	93.44 ± 0.84 *
LDSK95	DCM	0.50 ± 0.12 *	3.48 ± 0.28 *	9.86 ± 0.89 *
	EA	8.69 ± 0.29 *	12.31 ± 0.44 *	20.47 ± 1.14 *
	H_2_O	21.68 ± 0.91	23.30 ± 0.72 *	42.03 ± 0.58
LDSD50	DCM	30.10 ± 0.79 *	36.11 ± 1.06 *	58.03 ± 1.37 *
	EA	22.55 ± 0.63	26.48 ± 0.64	48.00 ± 0.87
	H_2_O	23.23 ± 1.08	26.24 ± 0.81	47.24 ± 1.12
LDSD95	DCM	24.59 ± 0.76	26.49 ± 1.14	47.21 ± 1.06
	EA	22.94 ± 0.65	28.02 ± 0.87	47.42 ± 1.09
	H_2_O	23.30 ± 0.47	32.95 ± 0.49	42.53 ± 1.11

Results were expressed as mean ± standard deviation (SD) values (*n* = 3). * Significant difference was detected in all fractions of same concentrations (*p* ≤ 0.05). % Inhibition demonstrated the maximum % inhibition of 2-DR degradation found for each concentration. LDSK50-DCM: fractions of *L. domesticum* skin was extracted with 50% aqueous ethanol and partitioned with dichloromethane; LDSK50-EA: fractions of 50% ethanol extract of the *L. domesticum* skin followed by ethyl acetate (EA) fractionation; LDSK50-H_2_O: The aqueous phase product when the *L. domesticum* skin was extracted with 50% aqueous ethanol and partitioned with EA; LDSK95-DCM: fractions of *L. domesticum* skin was extracted with 95% ethanol and partitioned with dichloromethane; LDSK95-EA: fractions of 95% ethanol extract of the *L. domesticum* skin followed by EA fractionation; LDSK95-H_2_O: the aqueous phase product when the *L. domesticum* skin was extracted with 95% ethanol and partitioned with EA; LDSD50-DCM: fractions of *L. domesticum* seed was extracted with 50% aqueous ethanol and partitioned with dichloromethane; LDSD50-EA: fractions of 50% ethanol extract of the *L. domesticum* seed followed by EA fractionation; LDSD50-H_2_O: The aqueous phase product when the *L. domesticum* seed was extracted with 50% aqueous ethanol and partitioned with EA; LDSD95-DCM: fractions of *L. domesticum* seed was extracted with 95% ethanol and partitioned with dichloromethane; LDSD95-EA: fractions of 95% ethanol extract of the *L. domesticum* seed followed by EA fractionation; LDSD95-H_2_O: the aqueous phase product when the *L. domesticum* seed was extracted with 95% ethanol and partitioned with EA.

The antioxidant capacity of 12 *L. domesticum* fractions determined by PCL and deoxyribose assays was summarized in Table 3.

**Table 3:** The antioxidant capacity of 12 *L. domesticum* fractions determined by photochemiluminescence (PCL) and deoxyribose assays.

Fractions		ACL	ACW	Deoxyribose
		Trolox eqv.(nmole) Mean ± SD	Ascorbic eqv.(nmole) Mean ± SD	Inhibition (%) Mean ± SD
LDSK50	DCM	1.030 ± 0.198	4.180 ± 0.211	43.94 ± 1.03
	EA	6.625 ± 0.445	54.660 ± 1.413	42.70 ± 0.86
	H_2_O	1.845 ± 0.007	98.733 ± 2.516	93.44 ± 0.84
LDSK95	DCM	0.380 ± 0.057	0.345 ± 0.007	9.86 ± 0.89
	EA	1.750 ± 0.057	8.350 ± 0.072	20.47 ± 1.14
	H_2_O	1.195 ± 0.120	9.910 ± 0.144	42.03 ± 0.58
LDSD50	DCM	0.795 ± 0.078	−0.065 ± 0.120	58.03 ± 1.37
	EA	1.257 ± 0.060	5.410 ± 0.240	48.00 ± 0.87
	H_2_O	0.525 ± 0.035	2.073 ± 0.101	47.24 ± 1.12
LDSD95	DCM	0.965 ± 0.050	1.513 ± 0.050	47.21 ± 1.06
	EA	1.200 ± 0.085	6.880 ± 0.028	47.42 ± 1.09
	H_2_O	0.635 ± 0.050	1.105 ± 0.007	42.53 ± 1.11

ACL (antioxidant capacity in lipid phase) tested at 10 µg of sample; ACW (antioxidant capacity in water phase) tested at 100 µg of sample; Deoxyribose assay tested at 2.0 mg/mL of sample; Results were expressed as mean ± standard deviation (SD) (*n* = 3); The bold characters demonstrated the maximum value of each assay. LDSK50-EA: fractions of 50% ethanol extract of the *L. domesticum* skin followed by ethyl acetate (EA) fractionation; LDSK50-H_2_O: The aqueous phase product when the *L. domesticum* skin was extracted with 50% aqueous ethanol and partitioned with EA; LDSK95-DCM: fractions of *L. domesticum* skin was extracted with 95% ethanol and partitioned with dichloromethane; LDSK95-EA: fractions of 95% ethanol extract of the *L. domesticum* skin followed by EA fractionation; LDSK95-H_2_O: the aqueous phase product when the *L. domesticum* skin was extracted with 95% ethanol and partitioned with EA; LDSD50-DCM: fractions of *L. domesticum* seed was extracted with 50% aqueous ethanol and partitioned with dichloromethane; LDSD50-EA: fractions of 50% ethanol extract of the *L. domesticum* seed followed by EA fractionation; LDSD50-H_2_O: The aqueous phase product when the *L. domesticum* seed was extracted with 50% aqueous ethanol and partitioned with EA; LDSD95-DCM: fractions of *L. domesticum* seed was extracted with 95% ethanol and partitioned with dichloromethane; LDSD95-EA: fractions of 95% ethanol extract of the *L. domesticum* seed followed by EA fractionation; LDSD95-H_2_O: the aqueous phase product when the *L. domesticum* seed was extracted with 95% ethanol and partitioned with EA; Eqv. means equivalent.

Regarding results demonstrated in Table 3, the *L. domesticum* fractions that exhibited the greatest antioxidant activity by PCL and deoxyribose assays were LDSK50-EA and LDSK50-H_2_O. These two fractions were classified as active fractions and selected for further study on their DNA-protective property against H_2_O_2_.

### 3.2. Antioxidative DNA Damage Activity of LDSK50-EA and LDSK50-H_2_O on TK6 Cells

To investigate the antioxidative activity of LDSK50-EA and LDSK50-H_2_O in protection of DNA damage, the TK6 cells were separately pre-treated with these two fractions at 25, 50, 100, and 200 µg/mL concentrations for 24 h prior to H_2_O_2_ induction. Treatments of TK6 cells with LDSK50-EA and LDSK50-H_2_O at these assigned doses for 24 h did not exhibit an inhibitory effect on cell growth rates. Results demonstrated in Table 4 indicated the percentage of TK6 living cells prior to H_2_O_2_ exposure (pre-H_2_O_2_) and after H_2_O_2_ exposure (post-H_2_O_2_) with different concentrations of LDSK50-EA and LDSK50-H_2_O fractions. In this study, any concentrations that produced cell viability of less than 70% were discarded in order to distinguish the oxidative effect from the cytotoxic effect.

**Table 4:** Percentage of living cells of pre- and post-H_2_O_2_ induction following treatments of TK6 cells with LDSK50-EA and LDSK50-H_2_O measured by the trypan blue exclusion method.

* LDSK50-EA			* LDSK50-H_2_O		
Concentration (µg/mL)	TK6 Viability (%)		Concentration (µg/mL)	TK6 Viability (%)	
	Pre-H_2_O_2_	Post-H_2_O_2_		Pre-H_2_O_2_	Post-H_2_O_2_
0	97.52 ±2.50	95.02 ± 6.80	0	97.52 ± 2.50	95.02 ± 6.80
25	98.15 ± 3.21	92.95 ± 4.37	25	98.72 ± 2.22	93.50 ± 5.73
50	99.57 ± 0.75	96.38 ± 6.28	50	97.24 ± 2.45	93.53 ± 5.29
100	97.62 ± 2.86	98.24 ± 1.94	100	97.70 ± 3.98	95.98 ± 6.97
200	97.76 ± 2.72	94.33 ± 4.27	200	98.89 ± 1.92	92.47 ± 7.96

* Results were expressed as means ± standard deviation (SD) (*n* = 3). LDSK50-EA: fractions of 50% ethanol extract of the *L. domesticum* skin followed by ethyl acetate (EA) fractionation; LDSK50-H_2_O: the aqueous phase product when the *L. domesticum* skin was extracted with 50% aqueous ethanol and partitioned with EA.

**Figure 2:**
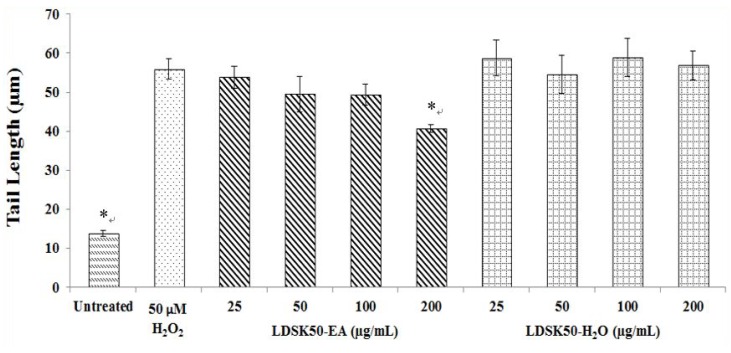
Tail lenght (TL, µm) values measured in pre-treated TK6 cells with LDSK50-EA (fractions of 50% ethanol extract of the *L. domesticum* skin followed by ethyl acetate (EA) fractionation) and LDSK50-H_2_O (the aqueous phase product when the *L. domesticum* skin was extracted with 50% aqueous ethanol and partitioned with EA) fractions followed by H_2_O_2_ damage induction by comet assay. Results were expressed as means ± standard deviation (SD) (*n =* 3). * Significant difference was detected from 50 µM H_2_O_2_ treatment groups at *p ≤* 0.05.

**Figure 3:**
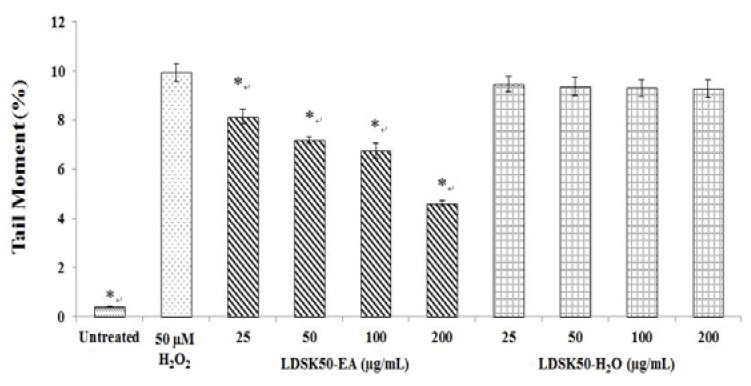
Tail moment (TM, %) values measured in pre-treated TK6 cells with LDSK50-EA (fractions of 50% ethanol extract of the *L. domesticum* skin followed by ethyl acetate (EA) fractionation) and LDSK50-H_2_O (the aqueous phase product when the *L. domesticum* skin was extracted with 50% aqueous ethanol and partitioned with EA) fractions followed by H_2_O_2_ damage induction by comet assay. Results were expressed as means ± standard deviation (SD) (*n =* 3). * Significant difference was detected from 50 µM H_2_O_2_ treatment groups at *p ≤* 0.05.

Results detected by comet or SCGE assay revealed that treatment of 50µM H_2_O_2_ for 5 min produced DNA damage (% TM, Figure 3) in TK6 cells at about 10-fold greater than untreated cells. Interestingly, this DNA damage could be prevented by pre-treating the TK6 cells with LDSK50-EA at 25, 50, 100, and 200 µg/mL for 24 h. The effect was found to be in a dose-dependent manner. The highest DNA preventive effect was found at 200 µg/mL concentration. In contrast, the LDSK50-H_2_O fraction exhibited a slight inhibitory effect on oxidative DNA damage when tested at similar concentration ranges. The DNA protective effect against H_2_O_2_ of LDSK50-H_2_O was indicated by a reduction in TL (Figure 2) and TM (= distance between the centre of gravity of the head to the centre of gravity of the tail) × (tail DNA intensity/total comet DNA intensity) (Figure 3) damage parameters in comparison to cells treated with H_2_O_2_ alone.

**Figure 4:**
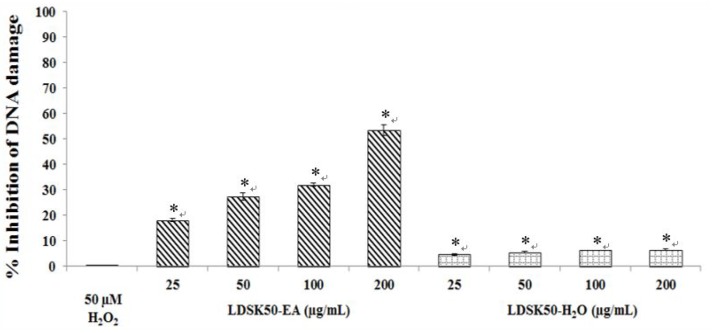
Inhibitory effect of LDSK50-EA (fractions of 50% ethanol extract of the *L. domesticum* skin followed by ethyl acetate (EA) fractionation) and LDSK50-H_2_O (the aqueous phase product when the *L. domesticum* skin was extracted with 50% aqueous ethanol and partitioned with EA) on H_2_O_2_-induced DNA damage in TK6 cells by comet assay. Results were expressed as means ± standard deviation (SD) (*n =* 3). * Significant difference was detected from 50 µM H_2_O_2_ treatment groups at *p ≤* 0.05.

The inhibitory oxidative DNA damage effect of LDSK50-EA and LDSK50-H_2_O in TK6 cells against H_2_O_2_ induction was illustrated in Figure 4. The results were calculated in percentage inhibition of DNA damage. The concentration-dependent DNA protective effect was found in LDSK50-EA by 18.04% ± 0.66%, 27.29% ± 1.37%, 31.99% ± 0.68%, and 53.47% ± 1.99% for 25, 50, 100, and 200 µg/mL, respectively. For LDSK50-H_2_O, a slight DNA protective activity was found and was not significantly different compared among four concentrations tested. The % inhibition of DNA damage of LDSK50-H_2_O was exhibited from low to high by 4.66% ± 0.36%, 5.59% ± 0.36%, 6.28% ± 0.06%, and 6.54% ± 0.25% for 25, 50, 100, and 200 µg/mL, respectively (Table 5).

**Figure 5:**
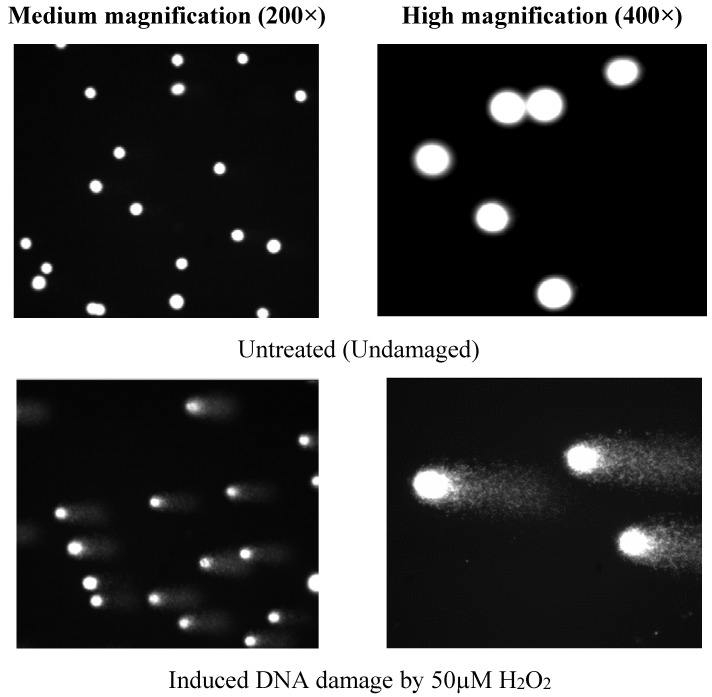

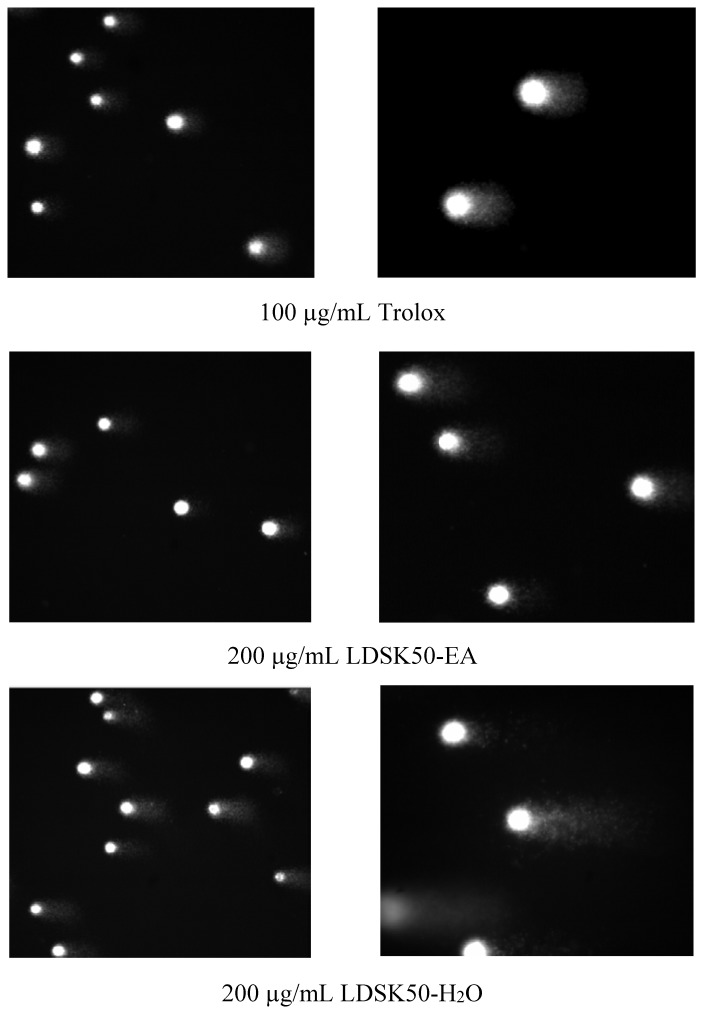
The comet images of TK6 cells (from top to bottom) of the following treatments: control or untreated (undamaged DNA), 50 µM H_2_O_2_, 100 µg/mL Trolox (positive control), 200 µg/mL LDSK50-EA, and 200 µg/mL LDSK50-H_2_O. Cells were stained with ethidium bromide and taken by fluorescence microscope at medium (200×) and high (400×) magnification. LDSK50-EA: fractions of 50% ethanol extract of the *L. domesticum* skin followed by ethyl acetate (EA) fractionation; LDSK50-H_2_O: the aqueous phase product when the *L. domesticum* skin was extracted with 50% aqueous ethanol and partitioned with EA.

Fluorescence images of comet TK6 cells evaluated for the different treatment groups were demonstrated in Figure 5.

**Table 5:** DNA damage parameters including tail length (TL) and tail moment (TM) and % inhibitory effect on DNA damage of LDSK50-EA and LDSK50-H_2_O in TK6 cells by comet assay.

Treatment	TL (µm)	TM (%)	DNA Damage Inhibition (%)
Untreated	13.80 ± 0.73 *	0.42 ± 0.02 *	NC
H_2_O_2_ 50 μM	55.97 ± 2.56	9.93 ± 0.37	0.00 ± 0.00
LDSK50-EA 25 μg/mL	53.82 ± 2.82	8.14 ± 0.30 *	18.04 ± 0.66 *
LDSK50-EA 50 μg/mL	49.50 ± 4.56	7.21 ± 0.13 *	27.29 ± 1.37 *
LDSK50-EA 100 μg/mL	49.37 ± 2.67	6.75 ± 0.30 *	31.99 ± 0.68 *
LDSK50-EA 200 μg/mL	40.75 ± 1.01 *	4.61 ± 0.10 *	53.47 ± 1.99 *
LDSK50-H_2_O 25 μg/mL	58.72 ± 4.56	9.46 ± 0.33	4.66 ± 0.36 *
LDSK50-H_2_O 50 μg/mL	54.60 ± 4.58	9.37 ± 0.37	5.59 ± 0.36 *
LDSK50-H_2_O 100 μg/mL	58.94 ± 4.81	9.30 ± 0.35	6.28 ± 0.06 *
LDSK50-H_2_O 200 μg/mL	56.89 ± 3.72	9.28 ± 0.35	6.54 ± 0.25 *

Results were expressed as mean ± standard deviation (SD) (*n =* 3). * Significant difference was detected from 50 μM H_2_O_2_ treatment groups at *p* ≤ 0.05 (ANOVA). NC = not calculated; LDSK50-EA: fractions of 50% ethanol extract of the *L. domesticum* skin followed by ethyl acetate (EA) fractionation; LDSK50-H_2_O: the aqueous phase product when the *L. domesticum* skin was extracted with 50% aqueous ethanol and partitioned with EA.

### 3.3. Determination of Phytochemical Components in LDSK50-EA TLC

**Figure 6:**
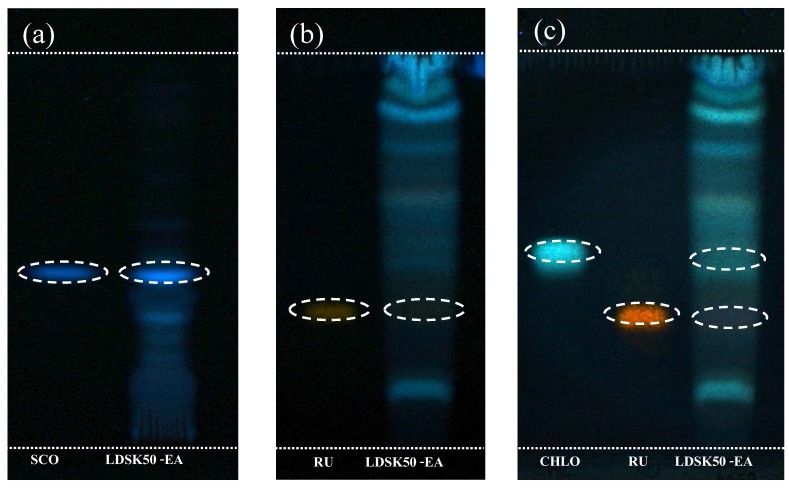
Thin Layer Chromatography (TLC) analysis of LDSK50-EA (fractions of 50% ethanol extract of the *L. domesticum* skin followed by ethyl acetate (EA) fractionation) fraction detected with Natural Product/polyethylene glycol (NP/PEG) spray reagent (366 nm) and against phytochemical standards including scopoletin (SCO), rutin (RU), and chlorogenic acid (CHLO). (**a**) toluene:ethyl acetate:formic acid (5:4:1) system; (**b**) and (**c**) ethyl acetate:formic acid:acetic acid:water (137:11:11:26) system.

LDSK50-EA was dissolved in absolute ethanol at a concentration of 100 mg/mL, and spotted in 10–20 µL aliquots onto silica gel F_254_ plates. The developing solvents were System 1: toluene:ethyl acetate:formic acid (5:4:1) and System 2: ethyl acetate: formic acid: acetic acid: water (137:11:11:26). After development, the plates were dried and sprayed with PEG reagent. Bands were visualized under ultraviolet (UV) detector at 366 nm and their *R*_f_ values were recorded and compared with three standard phytochemicals including scopoletin, rutin, and chlorogenic acid. TLC analysis of LDSK50-EA was shown in Figure 6. Under the detecting condition used in this study, the results clearly revealed a presence of scopoletin (*R*_f_ 0.44), rutin (*R*_f_ 0.34), and chlorogenic acid (*R*_f_ 0.49) in LDSK50-EA.

#### TPC Amount

The content of phenolic compounds was determined following the Folin-Ciocalteu method in comparison with standard gallic acid. The results are expressed in terms of mg GAE/mg sample extract. From our study, the TPC value for LDSK50-EA was 0.198 ± 0.001 mg GAE/mg extract.

#### TFC Amount

The content of flavoniod compounds was determined using the aluminum chloride colorimetric method in comparison with standard rutin and the results are expressed in terms of mg rutin equivalents (RE)/mg sample extract. This study showed that the TFC value of LDSK50-EA was 0.415 ± 0.005 mg RE/mg extract.

## 4. Discussions

### 4.1. PCL and Deoxyribose Assays of 12 Fractions of L. domesticum Extractions for Antioxidant Capacity against O_2_^−•^ and OH^•^ Radicals

It is well-accepted that ROS, such as O_2_^−•^, OH^•^, and H_2_O_2_ are highly reactive chemical species and can cause the oxidation of various biological molecules such as lipid, polypeptides, proteins, and DNA. Excess production and accumulation of ROS lead to oxidative stress, which can cause a number of diseases. In comparison with many other radicals, O_2_^−•^ is unreactive, but it can be converted into highly reactive species such as OH^•^, peroxyl (ROO^•^), and alkoxyl (RO^•^) radicals. Moreover, the dismutation of O_2_^−•^ can lead to the formation of H_2_O_2,_ which is the main source of OH^•^ through the Haber-Weiss and Fenton reactions [22]. In such conditions, the dietary intake of antioxidant compounds is needed to assist the body in neutralizing the free radicals and to remove the harmful effects of oxidative stress. Therefore, this study is aimed at evaluating the free radical scavenging activity of long-kong *L. domesticum* extracts.

PCL measures the potential antioxidant property of *L. domesticum* fractions by two different protocols, e.g., ACW and ACL, that measure the antioxidant capacity of the water- and lipid-soluble components, respectively [23,24]. The antioxidant property of compounds is quantified and expressed in equivalent concentration units of ascorbic acid and trolox equivalents for water- and lipid-soluble systems, respectively [25]. Our study found that all 12 *L. domesticum* fractions exhibited O_2_^−•^ scavenging activity at different degrees of activity for both ACL and ACW measurement systems. Results of the ACL demonstrated that the overall antioxidant capacity of the 12 fractions ranges from 0.380 to 6.625 nmol of trolox when all samples were tested at 10 µg/mL concentration. Among these, LDSK50-EA possessed the highest antioxidant activity with an equivalent to 6.625 nmol of trolox whereas other fractions exhibited slightly different antioxidant capacities. Interestingly, the antioxidant capacity of the ACW system indicated that the 50% ethanol extract of peel (LDSK50) still had a high antioxidant capacity. A wide range of antioxidant capacities of all fractions were found from −0.065 to 98.733 nmol of ascorbic acid. The highest antioxidant activity was found in the fraction of LDSK50-H_2_O (98.733 nmol of ascorbic acid), followed by LDSK50-EA (54.660 nmol of ascorbic acid).

Regarding the PCL results, they indicated that peels of *L. domesticum* fruits possessed higher O_2_^−•^ scavenging activity than seeds, particularly when extracted with 50% aqueous ethanol and partitioned with ethyl acetate (LDSK50-EA), which had high potential of both hydrophilic and lipophilic antioxidants. The results of ACL and ACW suggested that the O_2_^−•^ scavenger in LDSK50-EA fractions was of both polar and non-polar phytochemical groups. Furthermore, the OH^•^ radical scavenging activity of *L. domesticum* was also determined by the deoxyribose assay, another cell-free radical generating system. This assay monitored an inhibitory effect of *L. domesticum* fractions on 2-DR degradation by measuring the competition between 2-DR and sample fractions for the OH^•^ generated from the Fe^3+^/ascorbate/EDTA/H_2_O_2_ system. OH^•^ radicals formed in the solution were detected by their ability to degrade 2-DR into fragments that, on heating with TBA at a low pH, formed a pink chromogen [26,27]. The absorbance read at the end of the experiment was used for the calculation of the percentage inhibition of 2-DR degradation by the test samples [28,29].

When *L. domesticum* fractions were added to the reaction mixture, they removed OH^•^ from the sugar and prevented their degradation. The scavenging effect of *L. domesticum* fractions on OH^•^ was determined by monitoring the reduction of deoxyribose degradation. Results were expressed as % inhibition of 2-DR degradation. In the presence of *L. domesticum* fractions (0.5, 1.0, and 2.0 mg/mL concentration), a wide range of OH^•^ scavenging activity was found from 0.50 ± 0.12 to 93.44 ± 0.84. The LDSK50-H_2_O fraction has clearly presented to be the most effective inhibitor of the OH^•^ by exhibiting 93.44% ± 0.84% inhibition on 2-DR degradation. However, the wide range of % inhibition values among various *L. domesticum* fractions was possibly caused by their solubility character in the water, which was the solvent mainly used in the deoxyribose assay.

### 4.2. Antioxidative DNA Damage Activity of Two Active Fractions, LDSK50-EA and LDSK50-H_2_O, on TK6 Cells by Comet Assay

In the last two decades, the comet assay or SCGE has swiftly become one of the most popular methods in genetic toxicology. Its advantage is based upon a relatively fast, simple, and sensitive technique for the analysis of single-strand break (SSB), double-strand break (DSB), alkali-labile site (ALS) of DNA, and incomplete excision repair sites in eukaryotic individual cells [30,31]. Moreover, the comet assay has been extensively used for the investigation of the effects of antioxidants [32,33,34]. Among underlying principles, the alkaline (pH > 13) version of comet assay is superior for evaluating a broad spectrum of DNA lesions, and maximizes sensitivity for the detection of low levels of damage. Thus, it has been chosen as a useful general tool for monitoring DNA damage [31,35].

In this study, comet assay on TK6 cells was performed with the aim to evaluate the antioxidative DNA damage mechanism of LDSK50-EA against H_2_O_2_ induction. H_2_O_2_ is a direct non-radical reactive oxygen species. Though H_2_O_2_ itself is incapable of damaging DNA directly, it is the main source of OH^•^ through the Haber-Weiss and Fenton reactions [36,37]. The analysis of results obtained from the comet assay results was based on two major DNA damage parameters, e.g., the tail length (TL, in µm) and tail moment (TM, in %). However, there are comments concerning the use of these parameters since TL would reach a plateau value after migrating a certain distance but would still grow in intensity. Therefore, TM is generally considered the main representation of DNA damage [38,39].

The results of the comet assay from this study revealed that the treatment of H_2_O_2_ at 50 µM for 5 min produced DNA damage (% TM) in TK6 cells at about 10-fold greater than in untreated cells. This indicated that H_2_O_2_ clearly played the important role of oxidative DNA damage in TK6 cells. The geno-protective activity of LDSK50-EA and LDSK50-H_2_O in TK6 cells was found when cells were pre-treated with one of these two active fractions (25, 50, 100, and 200 μg/mL) for 24 h prior to exposure to H_2_O_2_. The DNA protective effect against H_2_O_2_ of LDSK50-EA and LDSK50-H_2_O was indicated by a reduction in TL and TM values in comparison to cells treated with H_2_O_2_ alone.

Interestingly, the H_2_O_2_-induced DNA damage in TK6 cells was prevented by LDSK50-EA pre-treatment at 25, 50, 100, and 200 µg/mL, in a dose-dependent manner. The highest DNA preventive effect was found at 200 µg/mL concentration with % DNA damage inhibition equal to 53.47 ± 1.99. However, the treatment of LDSK50-EA at a dose greater than 200 µg/mL (up to 250 µg/mL) caused a very little change in the % inhibitory effect, but induced high cytotoxicity. In contrast, the LDSK50-H_2_O fraction exhibited slight inhibitory oxidative DNA damage activity when tested at similar concentrations as LDSK50-EA. Nevertheless, the pre-treatment of cells with the highest dose (more than 1000 µg/mL) of LDSK50-EA did not induce a higher % inhibition effect.

### 4.3. Determination of Phytochemical Components in LDSK50-EA

TLC is a separation technique that has been generally used in chemistry to separate compounds in the mixture. It is generally agreed that TLC is most effective for the low-cost analysis of samples requiring minimal sample clean-up, or where TLC allows a reduction in the number of sample preparation steps. In this study, the TLC technique was used to detect the presence of phytochemicals in the LDSK50-EA active fraction. Following chromatogram development, the TLC plates were sprayed with various reagents, such as PEG reagent, to detect the phenolic compounds [40].

Phenolic compounds are characteristic of plants and as a group they are usually found as esters or glycosides rather than as free compounds. Current classification divides the broad category of phenolics into polyphenols and simple phenols, based solely on the number of phenol subunits present. Polyphenols possess at least two phenol subunits, including flavonoids, and those compounds with three or more phenol subunits are referred to as tannins (hydrolyzable and non-hydrolyzable) [41,42].

Under the natural product-PEG detecting condition, the results clearly revealed the presence of scopoletin (*R*_f_ 0.44), rutin (*R*_f_ 0.34), and chlorogenic acid (*R*_f_ 0.49) in LDSK50-EA. Subsequently, the TPC of LDSK50-EA was determined using Folin-Ciocalteu reagent to quantify the amount of phenolic compounds [20]. The results are expressed in terms of mg GAE/mg sample extract. From this study, the TPC value for LDSK50-EA was 0.198 ± 0.001 mg GAE/mg extract. At the same time, the TFC was determined using the aluminum chloride colorimetric method in comparison with standard rutin and the results are expressed in terms of mg RE/mg sample extract [43]. The results illustrated the TFC value of LDSK50-EA to be 0.415 ± 0.005 mg RE/mg extract. Overall, the results of determination of the phytochemical composition in the peel extract of *L. domesticum* fruits (LDSK50-EA) have shown that it was the major source of phenolic and flavonoid compounds. This finding was consistent with the earlier studies [44,45,46].

The data of this study warrant the good biological activities of LDSK50-EA, including antioxidant and antioxidative DNA damage activities. Its potent biological activities may be related to the occurrence of high potential phenolic and flavonoid substances. Many studies have demonstrated the antioxidant action of phenolic compounds, acting as terminators of free radical chains and as chelators of redox-active metal ions that are capable of catalyzing lipid peroxidation [47]. Similarly, the potent radical scavenging abilities of flavonoids could contribute by inhibiting lipid peroxidation and oxidation of the low density lipoprotein (LDL) [48].

A number of *in vitro* experiments have found that flavonoids exert a significant antioxidative ability due to the presence of the hydroxyl groups in the B ring of the basic flavonoid structure. It donates hydrogen atoms to radical reactions. The double-bond at position 2, 3 in conjugation with the 4-oxo-group in the C ring of the flavonoid structure, and the hydroxyl groups are capable of binding transition metal ions such as iron and copper. Hence, these contribute to the chelating ability of flavonoids. In the organism, the positive effect of flavonoids is exerted via several pathways. In addition to the antioxidative effect mentioned above, flavonoids also possess other antioxidative abilities, e.g., through the stimulation of antioxidative enzymes, and have vasodilating, anti-thrombotic, anti-inflammatory, and anti-apoptic effects. Moreover, flavonoids also exhibit anti-mutagenic abilities and can inhibit the bond of cancerogenic compounds to DNA [48,49].

## 5. Conclusions

The peel of *L. domesticum* fruits possessed higher O_2_^−•^ and OH^•^ scavenging activity than the seeds, particularly when extracted with 50% aqueous ethanol and partitioned with ethyl acetate (LDSK50-EA). This fraction had high potential of both hydrophilic and lipophilic antioxidants. Moreover, LDSK50-EA had a geno-protective effect by reduction of the DNA damage induced by H_2_O_2_ radicals, proven by comet assay in TK6 cells. This study generated new and updated information on the biological activity of extracts of long-kong fruits. It may lead to a discovery of new alternative sources of natural antioxidant and anti-genotoxic substances for the prophylaxis or treatment of free radical-related diseases as well as the development of the nutraceutical product industry.
